# Boron Supply Enhances Aluminum Tolerance in Root Border Cells of Pea (*Pisum sativum*) by Interacting with Cell Wall Pectins

**DOI:** 10.3389/fpls.2017.00742

**Published:** 2017-05-08

**Authors:** Xue Wen Li, Jia You Liu, Jing Fang, Lin Tao, Ren Fang Shen, Ya Lin Li, Hong Dong Xiao, Ying Ming Feng, Hai Xiang Wen, Jia Hua Guan, Li Shu Wu, Yong Ming He, Heiner E. Goldbach, Min Yu

**Affiliations:** ^1^Department of Horticulture, School of Food Science and Engineering, School of Life Science and Engineering, Foshan UniversityGuangdong, China; ^2^College of Resources and Environment, Huazhong Agricultural UniversityWuhan, China; ^3^State Key Laboratory of Soil and Sustainable Agriculture, Institute of Soil Science, Chinese Academy of SciencesNanjing, China; ^4^Plant Nutrition-Institute of Crop Science and Resource Conservation, University of BonnBonn, Germany

**Keywords:** Pea (*Pisum sativum*), aluminum toxicity, root border cells (RBCs), alkali-soluble pectin, chelator-soluble pectin, aluminum adsorption/desorption

## Abstract

Aluminum (Al) toxicity is the primary factor limiting crop growth in acidic soils. Boron (B) alleviates Al toxicity in plants, which is mainly considered to be due to the formation of Rhamnogalacturonan II-B (RGII-B) complexes, which helps to stabilize the cytoskeleton. It is unclear yet whether this is due to the increasing of net negative charges and/or further mechanisms. Kinetics of Al accumulation and adsorption were investigated using entire cells, cell wall and pectin of root border cells (RBCs) of pea (*Pisum sativum*), to reveal the mechanism of B in interacting with alkali-soluble and chelator-soluble pectin for an increased Al tolerance in RBCs. The results show that B could rescue RBCs from Al-induced cell death by accumulating more Al in the cell wall, predominately in alkali-soluble pectin. Boron also promotes Al^3+^ adsorption and inhibits Al^3+^ desorption from alkali-soluble pectin. Thus, more Al^3+^ is immobilized within the alkali-soluble pectin fraction and less in the chelator-soluble pectin, rendering Al^3+^ less mobile. Boron induces an increase of RG-II (KDO,2-keto-3-deoxyoctonic acid) content for forming more borate-RGII complexes, and the decrease of pectin methyl-esterification, thus creates more negative charges to immobilize Al^3+^ in cell wall pectin. The study provides evidence that abundant B supply enhances the immobilization of Al in alkali-soluble pectin, thus most likely reducing the entry of Al^3+^ into the symplast from the surroundings.

## Introduction

The role of essential element boron (B) is mainly related to mechanical properties in plants, and it plays a critical role in the structural integrity of the cell wall. In past studies, the function of B appears to be predominantly confined to the primary cell wall (Brown and Hu, 1997; Matoh and Kobayashi, 1998). Here it crosslinks the pectic polysaccharide rhamnogalacturonan II (RGII, O’Neill et al., 1996; Goldbach et al., 2007), the process that is beneficial to improve the physical and biochemical property of cell walls (Fleischer et al., 1998). Boron is found to bridge RGII during polysaccharide synthesis and secretion by gel electrophoresis (Chormova et al., 2014a,b), suggesting possible roles of B in polysaccharide synthesis. Supply of B has also been proved to alleviate Al toxicity in higher plants (LeNoble et al., 1996a,b; Stass et al., 2007; Zhou et al., 2015), possibly via cell wall since pectin is the common target of B deficiency and Al toxicity (Goldbach et al., 2007; Yang et al., 2011b). However, the mechanism of B and Al interactions in cell wall pectin are still largely unknown, with controversial reports on whether B supply reduces Al accumulation in root tips (Corrales et al., 2008; Yu et al., 2009b; Zhou et al., 2015).

Studies have shown that the binding of Al on the cell wall is a prerequisite for Al toxicity to plants, especially in the distal transition zone (Sivaguru and Horst, 1998; Tabuchi and Matsumoto, 2001; Horst et al., 2010; Kopittke et al., 2015). Roots restore quickly after Al is eluted by citrate from the apoplast (mainly from the cell wall; Liu et al., 2008). Increasing evidence suggests that cell wall pectin plays an important role in Al perception and response (Yang et al., 2008, 2011b; Horst et al., 2010). The negative charges of the pectic matrix in the cell wall are most likely the main adsorption sites of Al^3+^ (Zhang and Taylor, 1989; Blamey et al., 1990; Wang et al., 2004; Yang et al., 2011b). Hemicellulose also contributes significantly to Al adsorption in *Arabidopsis* and rice (Yang et al., 2008, 2011a). Generally, Al toxicity induces an increase of pectin contents in the cell wall, which provides more binding sites for Al ions, and thus the pectin content is positively correlated with Al sensitivity (Yang et al., 2008; Horst et al., 2010; Li et al., 2016). However, Al bound in different cell wall constituents (e.g., chelator-soluble or alkali-soluble pectin), might have contrasting fates, fixed in the cell wall or continuing to move into the symplast. Aluminum is more toxic within the symplast than in the apoplast, and Al resistant plants cope with Al toxicity by excluding Al from the root symplast (Kochian et al., 2004). Therefore, the cell-wall, especially its pectin fraction, seems to serve as a barrier for Al enter to the symplast of root cells. It is intriguing to better understanding how plant cells manage to balance these two opposing functions of the cell wall.

Root border cells (RBCs), previously termed as ‘sloughed off root cap cells’, are living cells programmed to separate from the periphery of roots into the rhizosphere and to surround the root apex (Hawes et al., 2000). They are considered to serve as protection for the root apex from abiotic and biotic stresses (Hawes et al., 2000), including Al toxicity (Yu et al., 2009a; Cai et al., 2013). RBCs protect the root and themselves from Al toxicity by immobilizing Al in alkali-soluble pectin instead of in chelator-soluble pectin (Yang et al., 2016), indicating that Al adsorption and desorption differs in pectins, which is crucial for the mobility of Al in the cell wall and toxicity of Al to plant cells. Strong fluorescence of B specific RGII is observed in RBCs, indicating a substantial requirement of B for cell wall integrity (Yu, unpublished data). It is pointed out that the accumulation of Al was higher in B supply roots than in B deficient roots despite B supply ameliorated Al toxicity to the roots of cucumber (Corrales et al., 2008). This finding has inspired us to investigate the roles of B in Al immobilization in alkali-soluble pectin of RBCs. We hypothesized that B supply might influence the binding strength of Al in the cell wall pectin, which impacts the kinetics of Al adsorption/desorption in cell wall.

Based on the analyses of Al content in RBCs and cell walls and kinetic assay of Al adsorption on and desorption from the cell wall/pectin, we investigate how B alleviates Al toxicity to RBCs of pea (*Pisum sativum*) by altering pectin properties.

## Materials and Methods

### Plant Materials

Seeds of pea (*Pisum sativum* L. Cv Zhongwan no. 5) were germinated under mist culture and RBCs were harvested as described previously (Yu et al., 2006, 2009a). Pea seeds were immersed in 5.25% sodium hypochlorite for 30 min, and thereafter rinsed six times with distilled water. Sterilized seeds were soaked in 0.5 mM CaCl_2_ solution (containing 0 or 50 μM H_3_BO_3_) for 10 h, and then evenly spread on the mesh screen of the mist watered germination facility. They were germinated at 24°C in 20 L plastic tanks with mist for 80 s every 8 min, which was produced from 2 mM CaCl_2_ solution containing 0 or 50 μM H_3_BO_3_. After 12 h, the solution was replaced by 0 or 1 mM AlCl_3_ solution (containing 2 mM CaCl_2_, 0 or 50 μM H_3_BO_3_, pH 4.5) for 24 h, respectively. RBCs were harvested by snipping root tips into a plastic beaker containing 0.5 mM CaCl_2_ solution (0 or 50 μM H_3_BO_3_) and stirring gently for 5 min. RBCs were pelleted at 4500 × *g* for 10 min after removing the root tips. Pellets were re-suspended in ultrapure water and centrifuged again. The rinsing procedure was repeated twice to yield purified RBCs subjected with/without B and Al pretreatment. The samples were used to analyze the Al content in cell wall component and the properties of cell wall pectins, and to assess the adsorption/desorption of Al ion in cell wall and pectins, or further Al exposure in solution to estimate Al uptake and accumulation in the cells and cell wall without Al pretreatment.

### Effect of B Supply on RBCs Viability and Al Adsorption in Cells and Cell Wall

Purified RBCs from mist culture (without Al pretreatment but with B pretreatment) were incubated in 0 or 100 μM Al solution (containing 0.5 mM CaCl_2_, pH 4.5) with 0 or 50 μM H_3_BO_3_ supply for 1 h. The cell number and cell viability was determined by trypan blue dye exclusion test (Yu et al., 2009a). Content of Al in the cells and cell walls were measured by colorimetric method using pyrocatechol violet and MES buffer (Zheng et al., 2004).

### Preparation of Cell Wall and Its Components

Cell wall materials were extracted according to Heim et al. (1991) and Hoson et al. (2003). Purified RBCs were broken by an ultrasonic cell crusher, and the homogenates were centrifuged at 4500 × *g* for 10 min. Then the precipitate was washed three times with 10 volumes of 80% ethanol and once with 10 volumes of methanol : chloroform mixture (1:1 [v/v]), followed by 10 volumes of acetone. The supernatant of each extract was discarded and the final pellet lyophilized. The dry powder was the crude cell wall fraction and was stored at 4°C for further analysis.

Pectin, hemicellulose and cellulose were extracted sequentially from the crude cell wall. Chelator -soluble pectin was first extracted with 0.5 M imidazole solution (pH 7.0); then alkali-soluble pectin was extracted with 50 mM Na_2_CO_3_ (containing 20 mM CDTA, 1,2-Diaminocyclohexane-*N,N,N′,N*′-tetraacetic acid monohydrate); hemicellulose was extracted with 4 M KOH (containing 0.1% NaBH_4_); and the final residue consists mainly of cellulose. Imidazole, Na_2_CO_3_ and CDTA in the pectin extracts were removed by dialysis (cutoff molecule weight 3 KD) in 0.5 mM CaCl_2_, and the processed pectin extracts were used to assess the kinetics of Al adsorption and desorption.

### Effect of B Supply on Al Content in Cell Wall Components

Al content in cell wall components was measured in RBCs exposed to B and Al in mist culture. Cell wall components were freeze-dried, and Al was extracted in 1 mL of 2 M HCl at room temperature for 24 h with occasional shaking. Al content in the supernatants was determined using ICP-AES (IRIS-Advantage, Thermo Elemental, MA, USA) after centrifugation at 4500 × *g* for 10 min.

### X-ray Photoelectron Spectroscopy (XPS) Studies

Purified RBCs from mist culture (without Al pretreatment but with B pretreatment) were treated incubated were treated in 0 or 100 μM Al solution (containing 2 mM CaCl_2_ and pH adjusted to 4.8 to avoid breakage of cells by lower pH) with 0 or 50 μM H_3_BO_3_ supply for 1 h. The RBCs were pelleted at 4500 × *g* for 10 min; then the supernatant was removed, the pellet blotted dry with filter paper; the RBCs was resuspended in 10 μL 95% (v/v) ethanol and blotted up by filter paper; and finally the RBCs were freeze – dried for analysis of surface Al content by X-ray photoelectron spectroscopy. All purified RBCs were vacuum- dried for at least 8 h before the measurement. RBCs samples were pressed onto plastic adhesive tape using a spatula to obtain a smooth surface for XPS measurement (VG multilab 2000 equipment Thermo VG scientific, East Grinstead, West Sussex, UK) using the Al Ka X-ray line of 1486.6 eV excitation energy at 300 W. To correct sample charges, high-resolution spectra were used as a reference by setting the C1s hydrocarbon peak to 284.6 eV. The background was subtracted. Data analysis was performed using Thermal Advantage software^1^. The ratios of atomic concentrations were calculated using the peak areas normalized on a basis of acquisition parameters and sensitivity factors proposed by the manufacturer.

### Al Adsorption Kinetics in Cell Wall Materials

Crude cell walls (without Al pretreatment but with B pretreatment) were used to assess the kinetics of Al adsorption /desorption. Cell wall powder was weighed into a 2 mL mini-column equipped with a filter at the bottom and equilibrated in 0 or 50 μM H_3_BO_3_ solution for 24 h at pH 4.5 at the flow rate of 0.1 mL⋅min^-1^. The adsorption solution consisted of 30 μM AlCl_3_ (pH 4.5) with or without 50 μM H_3_BO_3_. The flow rate of the solution was set to 0.2 mL⋅min^-1^ using a peristaltic pump. The fractions were collected at 10 min intervals until Al concentration in the adsorption solution was constant. Before the desorption, the Al treated cell walls were equilibrated in 0 or 50 μM B solution (pH 4.5) for 12 h in order to remove the Al remaining in the solution or not absorbed by the cell wall at the flow rate of 0.1 mL⋅min^-1^. Then cell walls were eluted by 0.5 mM CaCl_2_ and 2.5 mM CaCl_2_ solutions sequentially, at the flow rate of 0.2 mL⋅min^-1^. Aluminum was immediately determined by a colorimetric method using pyrocatechol violet and imidazole buffer (Ma et al., 1997). Finally, the amount of Al adsorbed onto or desorbed from the cell wall was calculated, and plotted against the time of adsorption / desorption.

### Al Adsorption and Desorption Kinetics in Pectin Solution

Five milliliters of each processed pectin extracts (without Al pretreatment but with B pretreatment) was pipetted into a dialysis bag (80 mm × 28 mm, cutoff molecular weight 3 KD) and equilibrated in 0 or 50 μM H_3_BO_3_ solution (pH 4.5) for 24 h. Then the dialysis bag was immersed in 500 mL solution of 30 μM AlCl_3_ (pH 4.5) for 24 h with gentle stir every 2 h. After the adsorption, the dialysis bag was placed into 100 mL deionized water for 2 s to remove surface Al^3+^. Finally, the dialysis bag was immersed in 500 mL desorption solution (containing 0.5 mM CaCl_2_, pH 4.5) for 24 h. The amount of Al adsorption or desorption per unit pectin was determined by measuring the difference of Al concentration in the adsorption or desorption solution colorimetrically using pyrocatechol violet (Ma et al., 1997).

### Analysis of Cell Wall Pectin

Contents of uronic acids and KDO were determined colorimetrically by hydroxydiphenyl (Blumenkrantz and Asboe-Hansen, 1973) and thiobarbituric acid (Karkhanis et al., 1978), respectively, in cell wall pectins extracted from RBCs exposed to B and Al in mist culture. Degree of pectin methyl-esterification (DM) was measured by a colorimetric method (Louvet et al., 2011). Pectin extracts (100 μL) were saponified by 50 μL 1.5 M NaOH solution for 30 min and then surplus alkali was neutralized by 50 μL 1.5 M HCl. The methanol produced from saponification was determined colorimetrically by a modification of the methods of Anthon and Barrett (2004) and Yang et al. (2008). Pectin DM was calculated as moles of methanol produced per mol galacturonic acid.

### Statistical Analysis

Means were compared using the Duncan’s multiple range test (*p* < 0.05). The percentages were transformed to arcsine in order to carry out the ANOVA analysis. All statistical analyses were performed using the statistical program SAS 9.13 (SAS Institute, Cary, NC, USA).

## Results

### Response of RBCs to Al Toxicity

**FIGURE 1:**
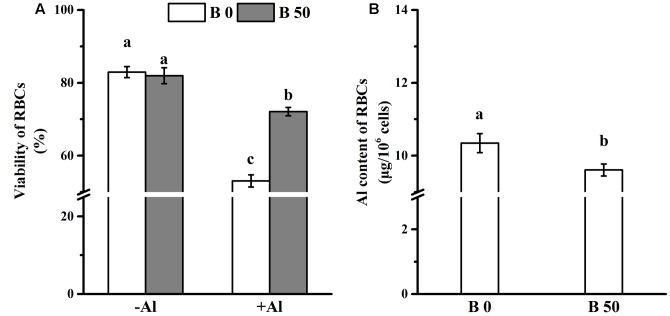
**Influence of B on cell viability and Aluminum accumulation of root border cells (RBCs).** RBCs was prepared by mist culture and treated with incubated in 0 or 100 μM AlCl_3_ solution (0.5 mM CaCl_2_, 0 or 50 μM H_3_BO_3_, pH 4.5) for 1 h. Viability of RBCs **(A)** was detected microscopically (magnification 100×) by the trypan blue exclusion test before and after Al treatment. Bars represent means ± SD, *n* = 20. After purification and desiccation, Al content of RBCs **(B)** was determined by colorimetric method using pyrocatechol violet. Bars represent means ± SD, *n* = 4. Different letters indicate significant difference at *p* < 0.05 (Duncan’s test).

Root border cells were harvested from the germinating pea seedling (2 days old). Cell viability was identical in RBCs with and without B supply in the absence of Al (**Figure 1A**). This indicates that B in the seeds can satisfy the B requirement for RBCs. The viability of RBCs decreased significantly after RBCs were exposed to 100 μM AlCl_3_ for 1 h *in vitro*. The viability was significantly higher in RBCs supplied with B (**Figure 1A**).

### Al Adsorption in RBCs and Its Distribution in the Cell Wall

**FIGURE 2:**
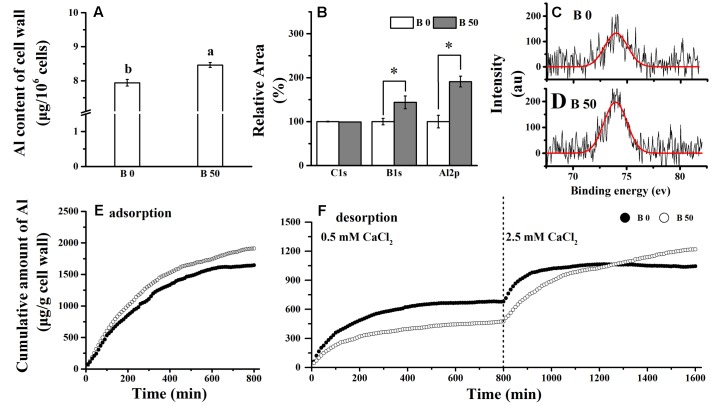
**Influence of B on Al content in cell wall of RBCs.** RBCs were treated with 100 μM AlCl_3_ solution for 1 h. Cell wall was extracted and dried. Cell wall Al content **(A)** was determined by colorimetric method using pyrocatechol violet. X-ray photoelectron spectroscopy (XPS) characterization of the chemical composition for C1s, B1s, and Al2p of the cell wall. The experiments were repeated three times and the relative content were calculated **(B)**. One of them of Al was compared **(C,D)**. The adsorption **(E)**/desorption **(F)** assay were performed on the cell wall with no Al added during the mist culture. The adsorption /desorption assay was repeated independently three times with the similar results and one set of results was presented. Bars represent means ± SD, *n* = 4. Different letters and ^∗^ indicate significant difference at *p* < 0.05 (Duncan’s test).

The adsorbed Al amounted to approximately 10.3 μg in 10^6^ RBCs without B supply; and it was significantly decreased by B supply (**Figure 1B**). However, Al content in cell walls of RBCs supplied with B was significantly higher than that without B supply (**Figure 2A**). XPS detects the surface elements down to a depth of 5–30 nm which is within the thickness of the cell walls. The results of XPS also showed that the cell wall Al content was increased when B was supplied (**Figures 2B–D**).

In the kinetics of adsorption/desorption of Al in cell wall materials. We found that amount of Al adsorption on cell wall material of B supplied RBCs was higher than that without B supply after 100 min (**Figure 2E**). In the process of desorption, less Al^3+^ was more desorbed by 0.5 mM CaCl_2_ solution while more desorbed by 2.5 mM CaCl_2_ solution in cell wall materials of RBCs with supplementary B than that without it. These results suggest that B not only promotes the accumulation of Al in the cell wall, but also enhances the strength of Al binding in the cell wall.

### Al Distribution in the Cell Wall Components

With regard to Al content in different cell wall components. We found that most of the Al (∼70%) was found in alkali-soluble pectin (**Table 1**). Al content in all the cell wall components increased after B supply, and it was extraordinarily higher in alkali-soluble pectin where Al content was 3.3-fold that without B supply. When the proportion of Al in the components was calculated, the results showed that B supply only increased the proportion of Al in the alkali-soluble fraction from 62.9 ± 3.0% to 74.2 ± 5.4%.

**Table 1:** Influence of B on Aluminum (Al) distribution in different cell wall components (μg/10^6^ cells); relative distribution is shown in brackets.

Treatment	The Al content of different cell wall components			
	Chelator-soluble pectin	Alkali-soluble pectin	Hemicellulose	Cellulose
B 0	0.18 ± 0.05a	0.94 ± 0.10b	0.32 ± 0.03b	0.06 ± 0.01a
	(11.9 ± 4.7%)	(62.9 ± 3.0%)	(21.5 ± 3.0%)	(3.8 ± 1.3%)
B 50	0.30 ± 0.09a	3.12 ± 0.16a	0.70 ± 0.06a	0.08 ± 0.03a
	(7.0 ± 2.0%)	(74.2 ± 5.4%)	(16.8 ± 3.4%)	(2.0 ± 0.7%)

*The cell wall components were extracted from RBCs exposed to 1 mM AlCl_3_ mist (2 mM CaCl_2_, 0 or 50 μM H_3_BO_3_, pH 4.5). The content of Al was determined by ICP-AES after extraction in 2 M HCl. Values (mean ±g SD, n = 3) with different letters indicate significant differences within the respective pectin fraction at p < 0.05 (Duncan’s test).*

### Pectin Properties

**FIGURE 3:**
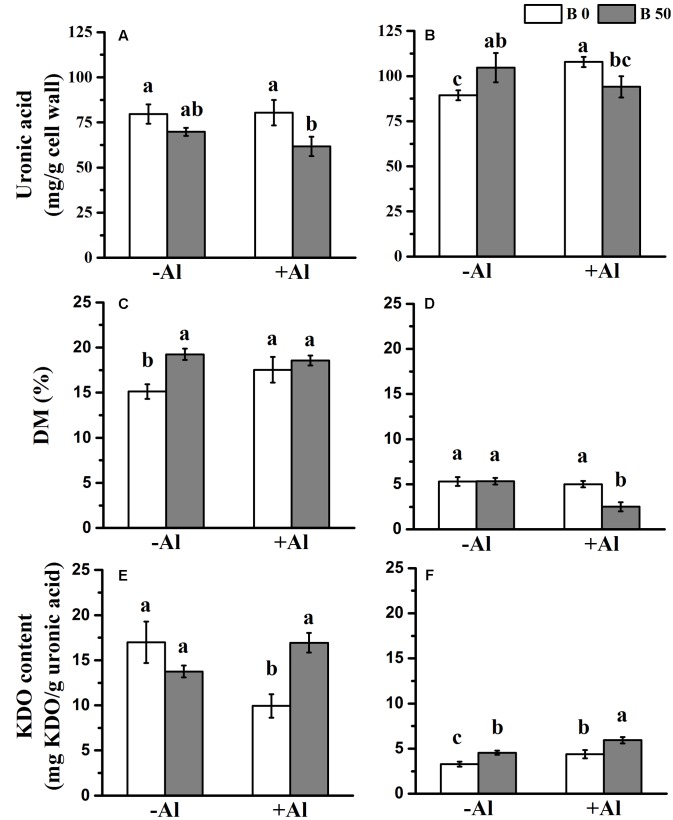
**Influence of B on the properties of chelator-soluble and alkali-soluble pectin in RBCs exposed to Al in mist culture (2 mM CaCl_2_, 0 or 50 μM H_3_BO_3_, pH 4.5).** Chelator-soluble pectin and alkali-soluble pectin were extracted by 0.5 M imidazole buffer (pH 7.0) and 50 mM Na_2_CO_3_ (containing 20 mM CDTA) solution, respectively. The uronic acid **(A,B)**, DM **(C,D),** and KDO contents **(E,F)** were determined in chelator-soluble pectin **(A,C,E)** and alkali-soluble pectin **(B,D,F)**. Bars represent means ± SD, *n* = 4. Different letters indicate significant difference at *p* < 0.05 (Duncan’s test).

In order to explore why B changes the distribution of Al in pectins, we analyzed the content of uronic acid, KDO, and DM. The result showed that alkali-soluble pectin was more abundant than chelator-soluble pectin in RBCs (**Figures 3A,B**). Al treatment only increased the content of alkali-soluble pectin of RBCs without B supply. In the presence of Al, both chelator-soluble and alkali-soluble pectin were reduced when B was supplied. The results indicate that under Al exposure, B supply does not promote an increase in alkali-soluble pectin as Al does. It rather decreases the content of both chelator-soluble pectin and alkali-soluble pectin.

The DM of chelator-soluble pectin was significantly higher than that of alkali-soluble pectin (ranged from 15 to 20% vs. 2 to 6%, respectively; **Figures 3C,D**). In the presence of Al, B application led to a significant decrease of DM in alkali-soluble pectin. The results show that B supply mainly promotes the decrease of DM in alkali-soluble pectin in the presence of Al.

The KDO content in chelator-soluble pectin was greater than that it was in alkali-soluble pectin (**Figures 3E,F**). B supply significantly increased KDO content in chelator-soluble pectin when combined with Al, and in alkali-soluble pectin with or without Al. Therefore, B supply increases KDO content in both chelator-soluble pectin and alkali-soluble pectin under Al toxicity.

### Al Adsorption/Desorption in Pectins

We investigated the effect of B on the Al adsorption and desorption in different pectin fractions. The results showed that alkali-soluble pectin adsorbed much more Al in comparison with chelator-soluble pectin (**Table 2**), by a factor of 5.3 to 9.2. B supply decreased Al adsorption in chelator-soluble pectin while promoting Al adsorption in alkali-soluble pectin. Most Al (68.5 ± 5.4%) adsorbed in chelator-soluble pectin was desorbed whereas only a minor proportion (8.5 ± 0.7%) was desorbed in alkali-soluble pectin. Boron promotes Al desorption from chelator-soluble pectin while inhibiting it from the alkali-soluble pectins.

**Table 2:** Influence of B on the adsorption/desorption of Al in chelator – soluble pectin and alkali – soluble pectin of RBCs of pea (*Pisum sativum*).

Treatment		The adsorption/desorption amount of Al^3+^ in pectin (μg⋅g^-1^)		Desorption rate (%)
		Adsorption of Al^3+^	Desorption of Al^3+^
Chelator – soluble pectin	B 0	547.5 ± 36.2a	379.1 ± 55.2a	68.5 (±5.4)b
	B 50	341.0 ± 13.4b	296.0 ± 9.3b	86.8 (±1.0)a
Alkali – soluble pectin	B 0	2922.4 ± 96.7b	249.1 ± 28.6a	8.5 (±0.7)a
	B 50	3134.0 ± 15.9a	38.6 ± 12.0b	1.2 (±0.4)b

*Cell wall pectin was extracted from RBCs at the absence of Al during the mist culture. The Al adsorption and desorption were performed by holding the pectin solution in dialysis bags in solution of 30 μM AlCl_3_ (pH 4.5) and 0.5 mM CaCl_2_ (pH 4.5) 24 h until equilibration, respectively. The concentration of Al in the solution was determined by colorimetric method using pyrocatechol violet. Values (mean ± SD, n = 3) with different letters indicate significant differences within the respective pectin fraction according to the Duncan’s test (p < 0.05).*

## Discussion

Root border cells are released from the root tip as individual cells or in small groups. They are viable components of the root system that play an important role in plant defense against heavy metals and Al toxicity. This is expected to depend predominately on the negative charges of cell wall pectins (Yang et al., 2016). Therefore, RBCs function as a barrier of Al to the roots and protect the pea plants from Al toxicity (Yu et al., 2009a). Here we demonstrate that B supply alleviates Al toxicity to RBCs by promoting Al immobilization in alkali-soluble pectin via changes in the properties of the cell wall pectins.

### Boron Alleviates Al Toxicity by Promoting Al Accumulation in Cell Wall of RBCs

Root border cells were sensitive to Al toxicity, which induced at loss of cell viability (Yu et al., 2006; Cai et al., 2011), that was mitigated by abundant B supply (**Figure 1A**). Previous study showed that B deficiency enhanced Al sensitivity in common beans (Stass et al., 2007) whereas B pretreatment alleviated Al toxicity to intact root or plants of cucumber and pea (Corrales et al., 2008; Yu et al., 2009b). These results demonstrate that B supply can reduce the toxicity of Al in roots and single cell population (RBCs). The accumulation of Al in root is significantly reduced by abundant B in common beans and peas (Stass et al., 2007; Yu et al., 2009b), but not in cucumber (Corrales et al., 2008). The results in this paper show that B also alleviates Al toxicity to RBCs of pea by reducing the Al accumulation (**Figure 1B**).

It has been assumed that Al binds preferentially on cell walls (Tabuchi and Matsumoto, 2001; Wang et al., 2014), and the binding of Al on the cell wall is toxic for cells as it modifies the physical and chemical properties of the cell wall, such as extensibility and ion-binding capacity (Delhaize et al., 1993; Ma et al., 2004; Horst et al., 2010). In this study, B supply decreased Al adsorption in RBCs and thus increased cell viability (**Figures 1A,B**), whereas Al accumulation in cell wall was increased (**Figures 2A–D**). It suggests that the accumulation of Al in cell wall rescues RBCs from Al toxicity by blocking the entrance of Al^3+^ to the cytosol. It is well known that the toxicity of Al in the symplast is more severe than in the apoplast, and could induce a burst of reactive oxygen species (Yamamoto et al., 2002; Huang et al., 2014), mitochondrial dysfunctions and cell death (Yamamoto et al., 2002; Panda et al., 2008; Li and Da, 2010). For RBCs, which are a single celled population, the accumulation of Al on the cell wall matches its function that is comparable to an exoskeleton surrounding the plant cell and providing both structural support and protection from biotic and abiotic stresses (Hamann, 2012). In the kinetics of Al^3+^ adsorption in cell walls, it is further confirmed that the cell walls of RBCs with B supply not only bind more Al^3+^, but also bind it more tightly: thus, a higher concentration of desorption solution (2.5 mM CaCl_2_ vs. 0.5 mM CaCl_2_) was required to desorb the Al^3+^ from the cell wall (**Figures 2E,F**).

### Alkali-Soluble Pectin is the Predominant Target of Al Accumulation that is Enhanced by B

**FIGURE 4:**
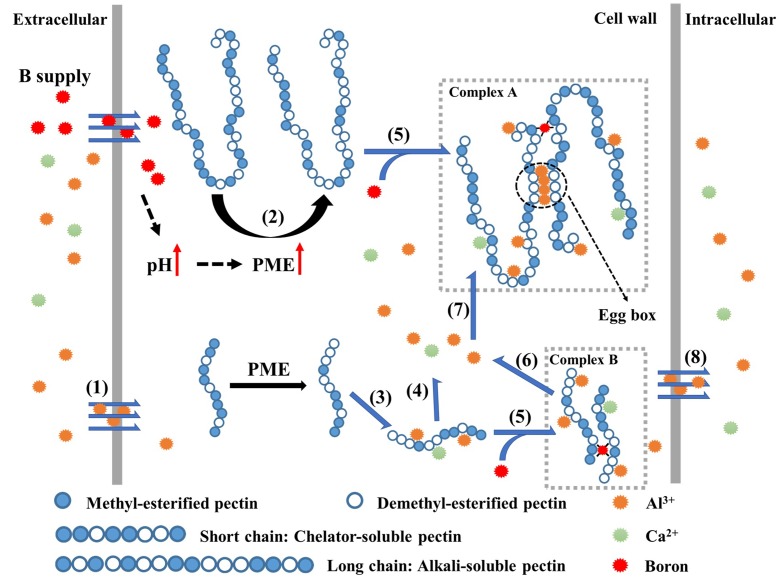
**Schematic kinetics of Al^3+^ adsorption/desorption on cell wall pectin.** At first, Al^3+^ enters the cell wall from extracellular space (1); B supply increases the apoplast pH and PME activity, promotes the demethylesterification of pectin (2), especially in alkali-soluble pectin (long chain). The demethylesterification of pectin facilitates the formation of “egg-box” in pectin chain with over 10 – consecutive unmethylated uronic residues, especially in alkali-soluble pectin where DM was lower; The difference of desorption rate in pectins results in higher desorption rate (4) of the adsorbed Al^3+^ (3) from chelator-soluble pectin and Al^3+^ tends to be fixed in alkali-soluble pectin (6), which is promoted by the formation of B-RGII dimers (complex B). Finally, less Al^3+^, from extracellular (1) or chelator-soluble pectin (4), can cross the plasmalemma after B supply (7).

Why does B increase Al bound in cell walls? It may depend on the feature of alkali-soluble pectin in Al immobilization. Most Al (∼70% in this study), is accumulated in alkali-soluble pectin (**Table 1**). Boron supply promoted the accumulation of Al in alkali-soluble pectin from 62.8 ± 3.0% to 74.2 ± 5.4% (**Table 1**). However, the accumulation of Al in chelator-soluble pectin was relatively decreased from 11.9 ± 4.7% to 7.0 ± 2.0% after B supply. This suggests that B changes the pattern of Al distribution in pectins. The increase of Al on alkali-soluble pectin may rely on the cross-linking of B with pectin (O’Neill et al., 1996; Goldbach et al., 2007) as Al^3+^ is a polyvalent cation, which rapidly and strongly binds to the negatively charged groups (Blamey et al., 1990). The cross-linking of B with RGII not only stabilizes the cytoskeleton, but also facilitates cation-binding on cell wall pectin by increasing the negative charges (**Figure 4** complex A and complex B, Fleischer et al., 1998; Chormova et al., 2014a). It may be the reason for an increased Al accumulation in the pectin (**Table 1**). The study of Al adsorption /desorption on different pectins types *in vitro* confirms that alkali-soluble pectin has a much higher binding capacity for Al^3+^ than chelator-soluble pectin does, and this property is further enhanced by B supply (**Table 2**). The desorption rate of Al^3+^ from alkali-soluble pectin was very low (8.5 ± 0.7 %), while it was much higher from chelator-soluble pectin (68.5 ± 5.4%), indicating that Al is easily desorbed from chelator-soluble pectin, in what seems to be a dynamic balance (**Figure 4** process 3 and process 4). It suggests that alkali-soluble pectin immobilizes Al while chelator-soluble pectin acts as a temporary “store” of Al. Therefore, most of Al will eventually be desorbed from chelator-soluble pectin (via progress 4 in **Figure 4**) and become bound to alkali-soluble pectin (via process 6 in **Figure 4**), or otherwise a minor portion reaches the cytoplasm (via the process 7 in **Figure 4**). B supply promotes both the desorption of Al^3+^ from chelator-soluble pectin and subsequently the immobilization of Al^3+^ in alkali-soluble pectin, therefore it inhibits Al entry into cytoplasm and alleviates Al toxicity to RBCs.

### Boron Promotes the Increase of RGII and the De-methylation of Alkali -Soluble Pectin

The Al-binding capacity of pectin depends on both pectin content and the degree of pectin methyl-esterification (Eticha et al., 2005a,b). Firstly, we investigated the pectin content and found that the uronic acid of alkali-soluble pectin was higher than that of chelator-soluble pectin under Al toxicity, indicating possibly more negative charges in alkali-soluble pectin. However, it was not increased when B was supplied (**Figures 3A,B**), suggesting that increase of Al immobilization on alkali-soluble pectin by B supply is unlikely to be caused by the increase of the pectin content. The analysis of pectin properties showed that DM was much lower in alkali-soluble pectin than it was in chelator-soluble pectin (**Figures 3C,D**). It means that the alkali-soluble pectin has more carboxyl groups than the chelator-soluble pectin, which facilitates the adsorption of Al on it (confirmed in **Table 2**). It is consistent with the finding that the higher DM pectin is more efficient in excluding the adsorption of Al (Mimmo et al., 2009), as showed in chelator-soluble pectin in this study, while lower DM facilitates Al adsorption in alkali-soluble pectin. Boron supply further decreases the DM in the alkali-soluble pectin, which, therefore, provides more binding sites and promotes Al adsorption in the alkali-soluble pectin fraction. However, Stass et al. (2007) show that B deficiency leads to an increase in unmethylated pectin in a root system, indicating the difference in DM change between tissue (root) and single cell population (RBCs). The role of B in promoting the demethylation of pectin in RBC may be related to the surface pH of roots. In another study, we found B supply could maintain a high H^+^ influx and surface pH in meristem and transition zone (Li unpublished data), where RBCs are sitting. PME has higher activity under higher apoplast pH (Downie et al., 1998; Denès et al., 2000; Jolie et al., 2009).

Boron mainly functions in cross-linking with pectin RGII to form RGII-B-RGII dimers (O’Neill et al., 1996). This process generates more negative charges (Scorei, 2012; Chormova et al., 2014b) and therefore may be the alternative reason for B in promoting Al adsorption on cell wall (**Figure 2A**) and pectin (**Table 1**). Boron supply promotes the increase of KDO content, a conserved residue of RGII, in both pectins under Al toxicity (**Figures 3E,F**). It indicates that more B bridging of RGII is formed under Al toxicity, which results in more negative charges. The increase of KDO also suggests changes of pectin properties which influences subsequently the kinetics of Al^3+^ adsorption. The adsorption of Al was enhanced in alkali-soluble pectin by B supply while it decreased in chelator-soluble pectin, and the reverse in Al desorption rate (**Table 2**). This was due to the decreased DM and the increased content of KDO by B supply in alkali-soluble pectin, while only KDO contents was increased by B supply in chelator-soluble pectin (**Figures 3E,F**). This indicates that B crosslinking with the pectin RGII promotes Al^3+^ immobilization in alkali-soluble pectins with a very low DM. The “egg-box” model may help to elucidate the higher charge density of alkali-soluble pectin. The structure of an “egg-box” may form in pectin with low methyl esterification having at least 10-consecutive unmethylated uronic acid residues which possess a higher charge density (Matoh, 2013; Levesque-Tremblay et al., 2015). Once Ca^2+^ (here Al^3+^) falls into the “dimple” of the pectin “egg-box” (Braccini and Pérez, 2001), it is tightly bound and forms structural pectin, being thus less mobile. B supply promotes de-methylesterification in alkali-soluble pectin, which possibly results in higher charge density and more “egg-boxes” to immobilize Al. In addition, heavy metals, e.g., Pb^2+^, and cationic chaperones, including polyhistidine and wall glycoproteins, facilitate B bridging with RGII (O’Neill et al., 1996; Ishii and Ono, 1999; Chormova et al., 2014b). Al^3+^ is a possible candidate to promote the B crosslinking with RGII, therefore the interaction of B and Al may lead to stronger Al immobilization in alkali-soluble pectin.

As summarized in the model above (**Figure 4**), we suggest that abundant B supply can promote the immobilization of Al in alkali-soluble pectin by promoting the de-methylesterification and increase of pectin RGII in alkali-soluble pectin. As a result, B reduces the entry of Al into the symplast, and thus alleviates Al toxicity to RBCs.

## Author Contributions

MY conceived the project and supervised the experiments; XL wrote the article and analyzed the data; XL, JL, JF, and JG performed most of the experiments; RS measured Al content and constructional suggestions to the research. YL, YF, HX, HW, and LT complemented the experiment. LW, YH, HG, and MY revised the manuscript.

## Conflict of Interest Statement

The authors declare that the research was conducted in the absence of any commercial or financial relationships that could be construed as a potential conflict of interest. The reviewer MZ and handling Editor declared their shared affiliation, and the handling Editor states that the process nevertheless met the standards of a fair and objective review.

## Footnotes

1 http://www.tainstruments.com
